# A blooming jellyfish in the northeast Atlantic and Mediterranean

**DOI:** 10.1098/rsbl.2010.0150

**Published:** 2010-04-07

**Authors:** P. Licandro, D. V. P. Conway, M. N. Daly Yahia, M. L. Fernandez de Puelles, S. Gasparini, J. H. Hecq, P. Tranter, R. R. Kirby

**Affiliations:** 1 Sir Alister Hardy Foundation for Ocean Science, Citadel Hill, Plymouth PL1 2PB, UK; 2 Marine Biological Association, Plymouth, UK; 3 Faculty of Sciences of Bizerte, Bizerte, Tunisia; 4 Instituto Espanõl de Oceanografía, COBaleares, Palma De Mallorca, Spain; 5 Laboratoire d'Océanographie de Villefranche—UPMC/CNRS, Villefranche/mer, France; 6 FRF-FNRS, MARE CENTER, University of Liège, Liège, Belgium; 7 University of Plymouth, Plymouth, UK

**Keywords:** climate, jellyfish blooms, *Pelagia noctiluca*, plankton, temperature

## Abstract

A long-term time series of plankton records collected by the continuous plankton recorder (CPR) Survey in the northeast Atlantic indicates an increased occurrence of Cnidaria since 2002. In the years 2007 and 2008, outbreaks of the warm-temperate scyphomedusa, *Pelagia noctiluca*, appeared in CPR samples between 45° N to 58° N and 1° W to 26° W. Knowing the biology of this species and its occurrence in the adjacent Mediterranean Sea, we suggest that *P. noctiluca* may be exploiting recent hydroclimatic changes in the northeast Atlantic to increase its extent and intensity of outbreaks. In pelagic ecosystems, Cnidaria can affect fish recruitment negatively. Since *P. noctiluca* is a highly venomous species, outbreaks can also be detrimental to aquaculture and make bathing waters unusable, thus having profound ecological and socio-economic consequences.

## 1. Introduction

Pelagic, true jellyfish (Cnidaria) form an abundant guild of top predators in marine ecosystems along with fish (Purcell & Arai 2001). Cnidaria vary in size from a few millimetres to a few metres, and they may be solitary (i.e. medusae of Hydrozoa, Scyphozoa and Cubozoa) or colonial (i.e. hydrozoan siphonophores), and have a life cycle that is either truly planktonic or that includes a benthic polyp stage (Boero *et al*. 2008). Cnidaria are important planktonic predators of fish larvae and their zooplankton food, so they can affect fisheries by bottom-up and top-down control of fish larval survival (Daskalov *et al*. 2007; Purcell *et al*. 2007).

Recent years' evidence indicates that Cnidaria have increased in abundance throughout the world's oceans and blooms (outbreaks of tens to hundreds of medusae per cubic metre) now occur more frequently in many seas (Purcell *et al*. 2007); these outbreaks have been attributed variously to alterations in the trophic structure of marine ecosystems owing to overfishing, and to hydroclimatic effects, since sea temperature can influence jellyfish life cycles and reproductive output (Purcell *et al*. 2007; Boero *et al*. 2008). The socio-economic effects of cnidarian outbreaks are not solely confined to pelagic fisheries, however. Since all Cnidaria possess stinging nematocysts, they are toxic and so can be detrimental to coastal aquaculture through damage to caged fish, and to tourism by curtailing bathing activities (Purcell *et al*. 2007).

Analysis of data collected by the continuous plankton recorder (CPR), the most temporally and spatially extensive plankton survey in the world, has revealed significant changes in northeast Atlantic plankton communities over the last two decades that appear to be related to hydroclimatic variability (Beaugrand 2004). While it has been suggested that hydroclimatic forcing has an important influence on the abundance of some North Sea cnidarian species (Lynam *et al*. 2005), the taxa in northeast Atlantic CPR samples are unknown, as their morphological identification is impossible.

Here, using molecular methods, we identify the Cnidaria in northeast Atlantic and northern North Sea CPR samples collected during 2007 and 2008 between 45° N to 58° N and 1° W to 26° W. To help understand the changes that have occurred, we compare our results to observations of Cnidaria in the western Mediterranean where long-term records are also available.

## 2. Material and methods

### (a) Plankton sampling in the northeast Atlantic

Data on cnidarian distributions and material for genetic analysis were obtained from the CPR survey (Batten *et al*. 2003). Each CPR sample represents the plankton from 3 m^3^ of water taken during 18 km of tow at an average depth of 7 m. Visual identification of cnidarian tissue and/or nematocysts was used as an index of presence and mapped for the period 1958–2007 using a 2° grid, where the nodes were calculated using the inverse-distance interpolation method (Isaaks & Srivastava 1989). A Kruskal–Wallis test was applied to detect the significance of changes in yearly, winter (November to April) and summer (May to October) averages for the periods 1958–1963, 1964–2001 and 2002–2007; these periods were identified by a cumulative sums analysis, which graphically detects local changes in a time series (Kirby *et al*. 2009). Data were then divided into two periods, 1958–2001 and 2002–2007, and the mean spatial distribution in each period, and the anomaly between them calculated.

### (b) Genetic analysis of Cnidaria in CPR samples

Genetic methods were used to identify Cnidaria in CPR samples. DNA was extracted from 34 samples of cnidarian tissue, collected from 15 separate CPR samples that were covered fully in cnidarian material, using standard protocols (Kirby *et al*. 2006). A 540-bp partial, mtDNA 16S rDNA sequence was then amplified by PCR using the primers of Cunningham & Buss (1993) and Schroth *et al*. (2002). The PCR involved an initial denaturation step of 94°C (1 min), 50 cycles of 94°C (1 min), 51°C (1 min) and 72°C (1 min) and a final extension of 72°C (10 min). The PCR products were sequenced using the forward amplification primer and their identity was established by comparison with GenBank. To help identification, a number of DNA sequences were also obtained from Cnidaria sampled by plankton net off Plymouth (England) and Stonehaven (Scotland). These sequences were obtained for both DNA strands (GenBank accession numbers EU999219–EU999230).

### (c) Records of Cnidaria from the Mediterranean

Cnidaria larvae (i.e. ephyrae) and adults were counted in plankton samples from the Gulf of Tunis (inshore and offshore), in the Bay of Calvi and the Bay of Villefranche (inshore only). The abundance of adult *Pelagia noctiluca* was also estimated visually in the Balearic and Alboran Seas and from the shore in the Bay of Calvi (table S1, electronic supplementary material). Records of scyphomedusae from the Mediterranean were used to help understand the seasonal progression of the main Cnidaria in northwest Atlantic CPR samples.

### (d) Temperature data

Sea surface temperature (SST) (1° grid) was obtained from the Hadley Centre, UK.

## 3. Results

**Figure 1.**
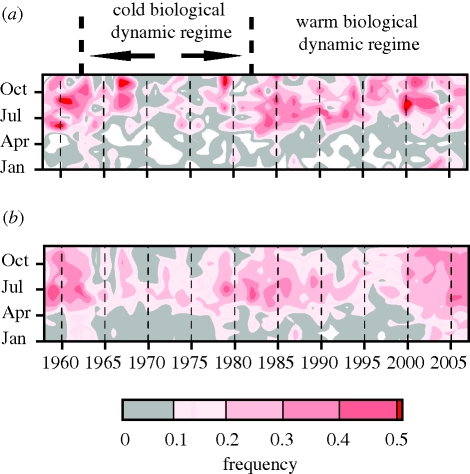
Average monthly frequency of Cnidaria in (*a*) North Sea and (*b*) northeast Atlantic CPR samples in 1958–2007.

**Figure 2.**
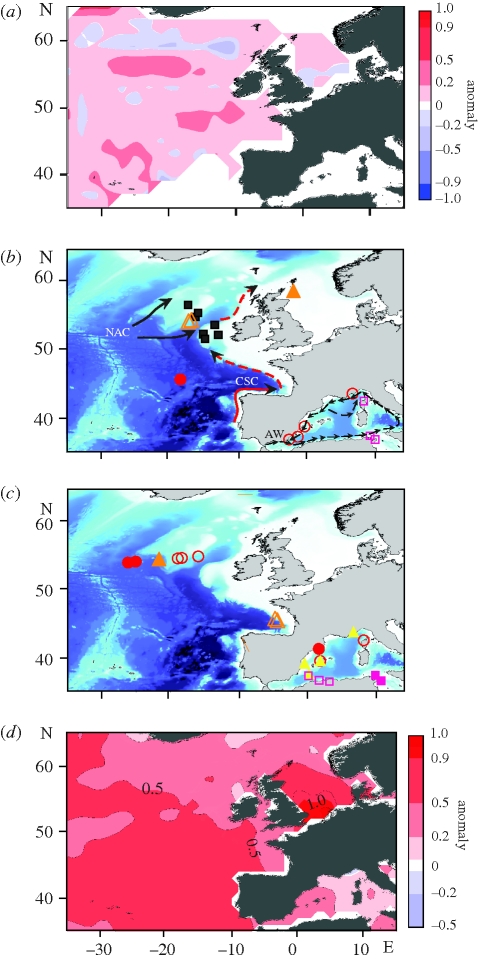
Cnidaria and sea surface temperature (SST) in the northeast Atlantic. (*a*) Map of the anomaly of the frequency of Cnidaria in CPR samples calculated as the difference in frequency between the periods 1958–2001 and 2002–2007. (*b,c*) Distribution of *P. noctiluca* outbreaks in the western Mediterranean and in CPR samples from the northeast Atlantic in (*b*) 2007 (black squares show records from Doyle *et al*. (2008)) and (*c*) 2008. Dashed black arrows indicate the progression of the Atlantic surface water (AW) stream in the western Mediterranean. Red and black arrows in the northeast Atlantic indicate continental slope current (CSC) and the North Atlantic Current (NAC), respectively. (*d*) Winter (January to March) SST anomaly (°C) of 2002–2008 versus 1958–2001. (*b*) Open pink square, Jan–Mar; open circle, Jul; filled circle, Sept; open triangle, Oct; filled square, Oct (see legend); filled triangle, Dec. (*c*) Filled pink square, Jan–Feb; open pink square, Feb; filled yellow triangle, May–Jun; open red circle, Jul; filled red circle, Aug; open orange triangle, Nov; filled orange triangle, Dec.

Analyses of CPR samples from the North Sea reveal an increase in frequency of Cnidaria since the early 1980s (figure 1*a*), coincident with a change from a cold to a warm hydroclimatic regime (Beaugrand 2004). In the northeast Atlantic the frequency of Cnidaria is greater during the winter months since 2002 than previously (*p* < 0.001), i.e. Cnidaria appear earlier in the year and persist for longer (figure 1*b*). The greatest increases in cnidarian abundance in this region occurred predominantly between 40° N to 58° N and 10° W to 30° W (figure 2*a*).

Genetic analysis of selected northeastern Atlantic CPR samples collected between 2007 and 2008 revealed four different species of siphonophore and the scyphomedusa *P. noctiluca*, which was the dominant Cnidaria (table S2, electronic supplementary material). Distributional data from the CPR and genetic analysis indicated outbreaks of *P. noctiluca* during 2007 at latitudes of 45° N, 54° N and 58° N in September, October and early December, respectively (figure 2*b*). In 2008, outbreaks of *P. noctiluca* occurred during summer and autumn at 54° N in the northeast Atlantic and at 45° N in the Bay of Biscay in November (figure 2*c*).

Observations of *P. noctiluca* in the western Mediterranean during 2007–2008 indicate that the seasonal progression of this species commenced in late autumn–winter in the southern region off the Tunisian coast and in the Bay of Calvi (high densities were maintained in the Bay of Calvi throughout the winter), ending in the Bay of Villefranche and in the Balearic and Alboran Seas in summer (figure 2*b,c*; table S3, electronic supplementary material).

## 4. Discussion

Jellyfish have increased in frequency in CPR samples from the northeast Atlantic since 2002, especially during winter. Molecular analyses of jellyfish in CPR samples reveal that *P. noctiluca* occurs over a large area coincident with where recent changes in Cnidaria are greatest (figure 2*a*). At this same time, outbreaks of *P. noctiluca* were reported in 2007 to cause mortalities of farmed fish in northeast Ireland and on the Scottish west coast (Doyle *et al*. 2008).

*Pelagia noctiluca* is a warm-temperate holoplanktonic scyphozoan and it is distributed widely from coastal to oceanic waters as far north as the northern North Sea (Hay *et al*. 1990). *Pelagia noctiluca* can acclimate to a wide range of temperatures (from less than 8°C to greater than 22°C in the Mediterranean, (Sandrini & Avian 1991), varying its metabolism to enhance the recruitment of young medusae (Morand *et al*. 1992), features that enable it to reproduce rapidly under favourable conditions to reach high densities the whole year round. Long-term records from the Mediterranean since the late nineteenth century reveal that outbreaks of this species, that tended to occur only once every 12 years and with 4 years duration before 1998, are now more frequent (Daly Yahia *et al*. 2010). As outbreaks of *P. noctiluca* appear to be associated with warm winters (Goy *et al*. 1989), the recent increase in western Mediterranean SST (up to 0.5°C increase since 2002 (figure 2*d*)) may explain their increasing frequency in this region. Warmer waters in the northeast Atlantic (up to 1°C increase in winter SST since 2002 (figure 2*d*)) may have influenced *P. noctiluca* similarly.

The seasonal occurrence of high densities of *P. noctiluca* in the western Mediterranean and northeast Atlantic also appears to be influenced by surface hydrography. In the western Mediterranean, the occurrence of *P. noctiluca* swarms follows the progression of the Atlantic surface water stream, which flows eastwards from the Atlantic through the Strait of Gibraltar along the North African coast (close to the Tunisian coast in winter) before circulating anticlockwise around the western Mediterranean basin (Pinardi & Masetti 2000) (figure 2*b*). In the northeast Atlantic, outbreaks of *P. noctiluca* appear to follow the progression of the North Atlantic Current (NAC) and the surface continental slope current (CSC), a northward branch of the Azores Current that flows along the eastern slope boundary of the European basin (Garcia-Soto *et al*. 2002; Pingree 2002) (figure 2*b*; figure S1, electronic supplementary material). Large-scale atmospheric patterns, i.e. the North Atlantic Oscillation (NAO), influence the strength of the NAC and CSC. In particular, during 2007–2008, the NAO was positive during winter and negative during summer, giving climatic conditions associated usually with an enhanced northward penetration of the NAC and CSC around Scotland into the North Sea (Garcia-Soto *et al*. 2002; Pingree 2002).

Predictions of global climate change suggest that the northeast Atlantic and North Sea will continue to warm (IPCC 2007). Owing to hydroclimatic change, warmer southern waters and species are now both recorded regularly further north penetrating into shelf regions (Beaugrand 2009; Graham & Harrod 2009). Increased advection and mixing of warmer and offshore waters into coastal shelf seas will also carry *P. noctiluca* and other jellyfish into environments with higher food resources, promoting jellyfish blooms. Outbreaks of *P. noctiluca*, along with other jellyfish, may therefore become more frequent and extend over a greater proportion of the year than previously. Any increase in jellyfish blooms may influence zooplankton production and fish recruitment to alter the pelagic food web. Since *P. noctiluca* is one of the most venomous species in waters around the British Isles (Mariottini *et al*. 2008), changes in its abundance may also have significant socio-economic effects.
